# Evaluating the Early Benefit of Quadrivalent HPV Vaccine on Genital Warts in Belgium: A Cohort Study

**DOI:** 10.1371/journal.pone.0132404

**Published:** 2015-07-06

**Authors:** Geraldine Dominiak-Felden, Corrado Gobbo, François Simondon

**Affiliations:** 1 Sanofi Pasteur MSD, Franchise Development Department, Lyon, France; 2 Sanofi Pasteur MSD, Diegem, Belgium; Georgetown University, UNITED STATES

## Abstract

Genital warts (GWs) are common, with about 5% to 10% of people having at least one episode in their lifetime. They develop about 2–3 months after infection with human papillomavirus (HPV) genotypes 6 and 11. The prophylactic quadrivalent HPV vaccine (qHPV), protects against HPV6/11 infections and diseases. In Belgium, HPV vaccines started to be reimbursed in 2007 and have been fully reimbursed since December 2008 for women 12 to 18 years old. This study aimed at evaluating the real-life benefit of qHPV vaccine introduction in Belgium on GWs by measuring both vaccine impact (VI) at a population level and the direct effect of the qHPV vaccine at an individual level (vaccine effectiveness (VE)), using data from a large sick-fund (MLOZ) reimbursement database. A first reimbursement for imiquimod (most common first-line GWs treatment in Belgium) was used as a surrogate for a first GWs episode; reimbursement of qHPV vaccine was used as surrogate for vaccination. VI was estimated by comparing the incidence of GWs before and after qHPV vaccine introduction in Belgium (ecologic evaluation). VE was assessed by comparing GWs incidences in vaccinated vs. unvaccinated women, among women eligible for HPV vaccination. VI was evaluated in 9,223,384 person-years. Overall, GWs incidence rates decreased significantly between the pre- and post-vaccination periods (-8.1% (95% CI: -15.3; -0.3) for men and women aged 18–59 years. This decrease was highest in women targeted by the HPV vaccination programme (-72.1% (95% CI: -77.9; -64.7) in women aged 16–22 years, with a 43% vaccine uptake in 2013). A significant decrease was also observed in men aged 16-22 years (-51.1%, 95%CI: -67.6; -26.2), suggesting herd-protection. VE was evaluated in 369,881 person-years. Age-adjusted VE for fully vaccinated women was 88.0% (95% CI: 79.4; 93.0). VE was higher when the first dose was given younger and remained high for over 4 years post-vaccination in all ages. High VI and VE of the qHPV vaccine were observed in a real-life setting in Belgium.

## Introduction

Human papillomaviruses (HPV) are DNA viruses that can be sexually transmitted and cause infection in basal epithelial cells such as mucosal or skin membranes [1]. Among the more than 100 HPV types that have been identified, fewer than 20 are thought to be oncogenic and are classified as ‘high-risk’ [2]. Infection with high-risk HPV types, such as HPV16 and HPV18 is a necessary pre-condition for the development of some cancers, including cervical and anal cancers [3]. Infection with low-risk HPV can lead to the development of genital warts (GWs); HPV types 6 and 11 are responsible for about 90% of them [4–6].

Between 5% and 10% of people have at least one episode of GWs in their lifetime [7–9]. They tend to appear shortly after first sexual activity, their incidence being highest in those aged 20 and 24 years old [10–13]. They are considered as benign but unsightly, and their treatment can be long. As treatment does not cure the underlying infection, recurrences are frequent, requiring repetitive and, sometimes, painful interventions [7]. GWs are known to cause psychological distress and to have a detrimental impact on quality of life [14, 15].

Two prophylactic HPV vaccines exist, both protecting against HPV types 16 and 18 infections and related diseases. Additionally, the quadrivalent HPV vaccine (Gardasil, qHPV), protects against HPV types 6 and 11 infections. As GWs tend to occur shortly after HPV 6/11 infection, the earliest benefits from qHPV vaccination are expected to be seen on this endpoint [10, 16].

In Belgium, HPV vaccines started to be reimbursed in 2007 for certain cohorts in some regions by some sick funds. They have been fully reimbursed since December 2008 for all women aged 12 to 18 years.

MLOZ (*Mutualités Libres-Onafhankelijke Ziekenfondsen*; in English: National Union of Independent Sick Funds) is one of the three biggest sick funds in Belgium. It represents about 18% of the Belgian population with more than 2 million affiliates. To perform its mission, MLOZ maintains a database containing information on its affiliates’ reimbursements. The database includes information on reimbursements of qHPV vaccine and imiquimod, which is a first-line treatment for GWs in Belgium, used for about 70% of GWs episodes [17].

Although the results from randomized controlled trials have shown that the qHPV vaccine is highly efficacious in preventing HPV6/11 related GWs, it is important to evaluate its performance in a real-life setting to confirm that the high effect observed under ideal clinical trial conditions is seen under conditions of routine use [18]. This can be achieved either by assessing the impact of vaccination programmes at a population level (i.e. vaccine impact (VI)) or by assessing the direct protective effect of vaccine at an individual level (i.e. vaccine effectiveness (VE)) in a real-life setting [19]. Several studies evaluating the VI of national qHPV vaccination programmes in Europe and elsewhere have already been published [20–25]. The observed decrease in GWs incidence following qHPV vaccine introduction varied according to the country and the local implementation of vaccination programmes. However, because of the type of data required, very few studies have evaluated both VI and VE [21, 26].

This study aimed at evaluating the real-life benefit of the introduction of qHPV vaccine in Belgium on GWs by measuring both the VI at the population level and the VE at an individual level, using a large sick fund (MLOZ) reimbursement database.

## Materials and Methods

### Ethics

The study was performed respecting the established standards laid out in the Good Epidemiological Practice (GEP) guidelines as well as the principles of the Declaration of Helsinki. In addition, the study complied with the Belgium national ethics regulations. The study did not require ethics committee approval. The analysed data were already stored in an electronic database run by the MLOZ sick fund to record routine reimbursement for healthcare-related costs and managed by the MLOZ staff according to confidentiality clauses in line with data protection laws. The data had been anonymised and de-identified prior to transfer and analyses, thus no written informed consent was required. All people involved in the analyses complied with national ethics regulation and data protection laws.

### Study Design

A retrospective cohort study was conducted using the MLOZ reimbursement database. This database includes socio-demographic data, as well as reimbursement information on its affiliates, in particular information on qHPV vaccine and imiquimod reimbursement. This information can be linked at an individual level using unique identification numbers. Imiquimod is reimbursed at a specific price by MLOZ for GWs treatment, enabling its prescription for GWs to be distinguished from prescription for other indications, such as actinic keratosis, or small superficial basal cell carcinoma. In addition, a preliminary agreement from a sick fund physician is required prior to reimbursement. Hence, imiquimod agreements at this specific level of reimbursement were used as a surrogate endpoint for GWs.

### Definitions

A first GWs episode was defined as an agreement for a first prescription of imiquimod with a level of reimbursement specific for GWs; subsequent agreements were excluded. The date of agreement was considered as the date of GWs onset. The date of vaccine reimbursement was considered as the date of vaccination.

### Impact of Vaccination Programme at the Population Level (VI)

All women and men aged 16–59 years, affiliated to MLOZ were included in the VI evaluation. The overall study period was January 2006 to December 2013. Three sub-periods were defined according to different stages of the HPV vaccination programme implementation: pre-vaccination—2006; transitional– 2007–2008; and post-vaccination: 2009–2013.

VI was evaluated by comparing the GWs incidence rate (IR) before and after the qHPV vaccine introduction in Belgium (ecologic evaluation). Annual GWs IRs were computed as the number of first imiquimod agreements by birth cohort, gender and calendar year divided by the corresponding number of MLOZ affiliates on January 1^st^ each year.

In parallel, the cumulative qHPV vaccine uptake rate was computed by age and calendar year as the number of first qHPV vaccine reimbursements in women by birth cohort and calendar year, divided by the corresponding number of affiliated women on January 1st each year.

The change in GWs IRs between pre and post-vaccination periods was stratified by age and gender according to the likelihood of being targeted by the HPV vaccination programme: 1) population targeted (i.e. women aged 16 to 22 years); 2) untargeted population (i.e. women aged ≥23 years and all men), allowing to assess potential herd protection; 3) overall population (i.e. women and men, aged 16–59 years), allowing to assess the overall effect of the vaccination programme [19]. IRs between periods were compared using incidence rate ratios (IRR) and percentage changes.

Both age and gender are known to be associated with vaccine uptake and the risk of HPV infection. As the overall study period was long (2006–2013), a direct standardisation on age and gender was performed to adjust for any change in age and gender distributions in the MLOZ population that could affect the comparability of the GWs IRs between the study periods. The reference population used was the overall age/gender distribution of the MLOZ population throughout the whole study period in person-years. The main results were standardised on age and gender.

### Vaccine Effectiveness Evaluation

For the VE evaluation, the study population was restricted to women eligible for HPV vaccine reimbursement by MLOZ between 2007 and 2013, i.e. all affiliated women born between 1st January 1990 and 31st December 1997. Women were included either on 1 January 2007, or on the date of their affiliation to MLOZ, if they were ≥16 years or on the date of their 16th birthday. They were followed until one of the following events occurred, whichever came first: end of the study (i.e. 31 December 2013), date of their 24th birthday, date they left the sick fund or date of first imiquimod agreement. In the primary analysis, an individual was considered as protected by a given vaccine dose 30 days after the date of vaccine reimbursement, to be consistent with the definition used in the qHPV vaccine clinical trials. Women having had a qHPV vaccine reimbursement before the study started were considered as being vaccinated at the study start. A woman was considered to be fully protected, 30 days after all three doses of qHPV vaccine had been reimbursed, irrespective of the delay between each dose. Women who had been reimbursed for one or two doses, irrespective of the delay between the doses, were considered to be partially vaccinated. Adherence to vaccination schedule was defined as: reimbursement for second and third doses obtained no later than 2.5 and 12 months after the first dose, respectively, since the recommended vaccination schedule is three doses given at 0, 2, 6 months. A woman was considered to be unvaccinated at any given time if she had not had a qHPV vaccine reimbursement at that time, or if the reimbursement was dated less than 30 days previously.

The primary objective of the study was to estimate and compare the incidence of GWs between women fully vaccinated with qHPV vaccine and women who had not been vaccinated with either HPV vaccine (qHPV or bivalent HPV (bHPV) vaccine). In order to be conservative, women vaccinated with the bHPV vaccine, which contains HPV16 and 18 only, were excluded from the main analysis, although this vaccine is not expected to protect against HPV 6 and 11.

The secondary objectives were to compute the qHPV VE by number of doses (one, two, or three), adherence with the recommended vaccination schedule, time since vaccination and age at first dose (<15 years; 15–17 years; ≥18 years).

GWs IR by vaccination status was calculated as the number of agreements for a first imiquimod prescription divided by the number of days each woman contributed to each group (unvaccinated, vaccinated with 1, 2 or 3 doses), expressed in person-years, allowing the same women to contribute person-time to multiple dose categories. The IRR was calculated as the ratio of the GWs IRs between unvaccinated and either fully or partially vaccinated women. VE was calculated as: VE = 1-IRR*100 and was expressed as a percentage.

The impact of the following potential confounding factors, that were available in the MLOZ database, was tested: age; agreement for first HIV treatment; parity; parents’ socioeconomic status and region of residence. Factors were considered to be confounding if the difference between the crude and adjusted VE was ≥15%. Multivariable analysis was conducted using Poisson regression to calculate adjusted VE. Variables with p>0.10 were removed in stepwise manner.

Finally, two sensitivity analyses were conducted to increase the statistical power of the VE estimate. In the first, all women aged 16 to 23 years, were included, irrespective of their eligibility for vaccine reimbursement as defined by their birth cohort. Although not all women aged 23 years were eligible for vaccination, it was expected that this analysis would increase statistical power as the GWs incidence is high in this age group. In the second, women vaccinated with bHPV vaccine were included in the non-qHPV vaccinated group (i.e. in the comparison group), to increase the number of person-years in this group. This was based on the assumption that the bHPV vaccine has no effect on GWs as it is not expected to protect against HPV6 and 11. This assumption was tested and confirmed before merging the two groups, by comparing the GWs IR in women who had received three doses of bHPV vaccine with that in women who had not received any HPV vaccine.

All analyses were performed using Stata 12.0.

## Results

### Impact of Vaccination Programme at the Population Level (VI)

Overall the VI analyses included 907,047 individuals in 2006, increasing to 1,284,493 individuals in 2013, providing 9,223,384 person-years of follow-up. During this period, 8,090 new GWs episodes (4,533 for women and 3,557 for men) were recorded. Overall, the GWs IR decreased significantly between pre and post-vaccination periods (-8.1%, 95% CI: -15.3; -0.3) for men and women aged 18–59 years (Table 1). The largest reduction was observed in women aged 16 to 22 years, the population targeted by the qHPV vaccination programme; -72.1% (95% CI: -77.9; -64.7) (Table 1). In women aged 23 to 30 years, the GWs IR was also significantly reduced (-18.8%, 95% CI:-32.8; -1.8). In contrast, the GWs IR increased by 20.0% (95% CI: -4.4; 50.6) and 33.1% (95% CI: 4.6; 69.4) in women aged 31 to 40 years and 41 to 59 years, respectively.

**Table 1:** Impact of the HPV vaccination programme. Age- and gender-standardised genital warts incidence rates estimates in the pre-vaccination (2006), transitional (2007–2008) and post-vaccination (2009–2013) periods—individuals affiliated to the MLOZ sick fund (Belgium).

	Pre-vaccination	Transitional	Post-vaccination	Transitional vs. Pre-vaccination		Post- vs. pre-vaccination	
Population: gender (age, years)	Incidence per 100 000 (95% CI)	Incidence per 100 000 (95% CI)	Incidence per 100 000 (95% CI)	Incidence rate ratio (95% CI)	Percentage change (95% CI)	Incidence rate ratio (95% CI)	Percentage change (95% CI)
**Targeted population**							
Women (16–22)	257.25 (218.22; 301.23)	183.43 (161.65; 207.33)	71.49 (59.69; 84.95)	0.72 (0.59; 0.87)	-28.48 (-41.44; +12.66)	0.28 (0.22; 0.35)	-72.07 (-77.90; -64.72)
**Not targeted population**							
Men (16–22)	66.77 (48.32; 89.94)	43.96 (34.00; 55.93)	32.64 (24.15; 43.15)	0.66 (0.45; 0.97)	-34.04 (-55.15; -3.01)	0.49 (0.32; 0.74)	-51.06 (-67.56; -26.17)
Men (23–30)	123.37 (99.70; 150.98)	145.82 (128.60; 164.70)	141.31 (124.37; 159.92)	1.18 (0.98; 1.49)	+17.68 (-7.03; +48.94)	1.14 (0.90; 1.45)	+14.22 (-9.84; +44.70)
Men (31–40)	89.96 (73.35; 109.21)	93.45 (82.16; 105.86)	99.50 (87.84; 112.28)	1.03 (0.82; 1.10)	+3.27 (-17.97; 30.02)	1.10 (0.88; 1.38)	+10.09 (-12.37; +38.31)
Men (41–59)	43.53 (35.09; 53.39)	50.54 (44.46; 57.22)	55.21 (48.84; 62.17)	1.17 (0.92; 1.48)	+16.55 (-8.28; +48.11)	1.27 (1.00; 1.61)	+27.25 (+0.41; +61.27)
Women (23–30)	223.22 (190.36; 260.12)	220.11 (198.45; 243.49)	181.58 (161.95; 202.92)	0.99 (0.82; 1.19)	-1.33 (-17.86; +18.53)	0.81 (0.67; 0.98)	-18.75 (-32.75; -1.83)
Women (31–40)	90.53 (73.74; 110.00)	103.81 (91.80; 116.96)	108.81 (96.50; 122.25)	1.15 (0.91; 1.44)	+14.61 (-8.79; +44.01)	1.20 (0.96; 1.51)	+19.98 (-4.38; +50.55)
Women (41–59)	43.40 (34.76; 53.53)	55.21 (48.68; 62.37)	57.99 (51.29; 65.32)	1.27 (0.99; 1.62)	+26.76 (-0.53: +61.55)	1.33 (1.05; 1.69)	+33.09 (+4.59; +69.37)
Women (16–59)	113.62 (103.93; 123.96)	112.02 (105.67; 118.64)	92.21 (86.56; 98.12)	0.98 (0.89; 1.09)	-1.54 (-11.30; +9.29)	0.81 (0.73; 0.90)	-18.96 (-27.18; -9.81)
Men (16–59)	71.13 (63.68; 79.23)	75.59 (70.51; 80.95)	76.98 (71.84; 82.38)	1.06 (0.94; 1.21)	+6.25 (-6.48; +20.71)	1.08 (0.95; 1.23)	+8.14 (-4.79; +22.82)
**Overall population**							
Women and men (16–59)	92.02 (85.89; 98.48)	93.41 (89.34; 97.61)	84.52 (80.69; 88.49)	1.02 (0.94; 1.10)	+1.54 (-6.34; +10.08)	0.92 (0.85; 1.00)	-8.09 (-15.31; -0.26)

A significant decrease in GWs IR was also observed in men aged 16 to 22 years; (-51.1%, 95% CI: -67.6; -26.2) (Table 1). In contrast, the GWs IR increased by 10.1% (95% CI: -12.4; +38.3) in men aged 31 to 40 years and by 27.3% (95% CI: +0.4; +61.3) in men aged 41–59 years (Table 1).

**Fig 1:**
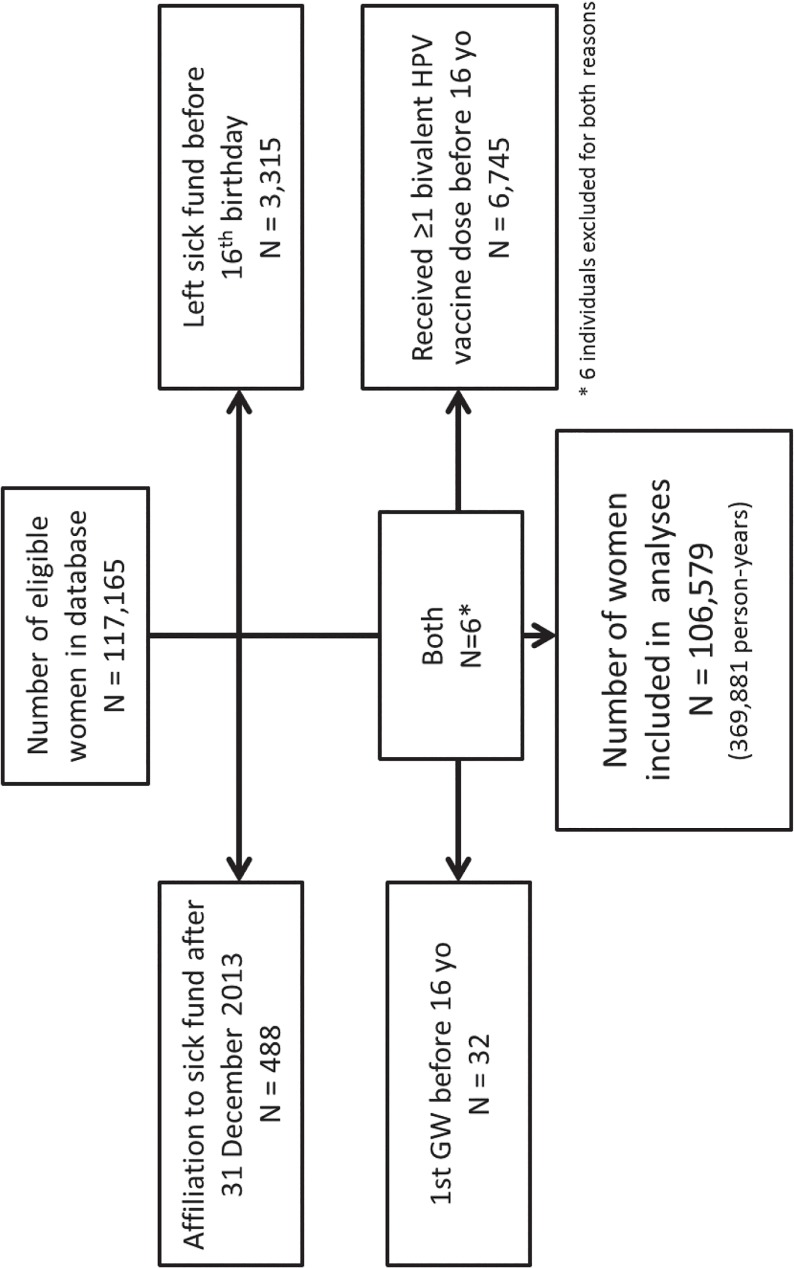
Incidence of genital warts per 100 000 individuals (dashed line: females; solid line: males) and qHPV vaccine uptake in individuals aged 16–22 years (A), 23–30 years (B), 31–40 years (C) and 41–59 years (D) affiliated to the MLOZ sick fund (Belgium) between 2006 and 2013 (standardised estimates).

The qHPV vaccine uptake (at least one dose) increased from 0% in 2006 to 48% in 2013 in 16–22 year-old women and from 0% in 2012 to 2% in 2013 in 23–30 year-old women (Fig 1). In men and women aged 16 to 22 years, the GWs IRs tended to decrease over time as vaccine uptake increased in women (Fig 1).

### Vaccine Effectiveness Evaluation

**Fig 2:**
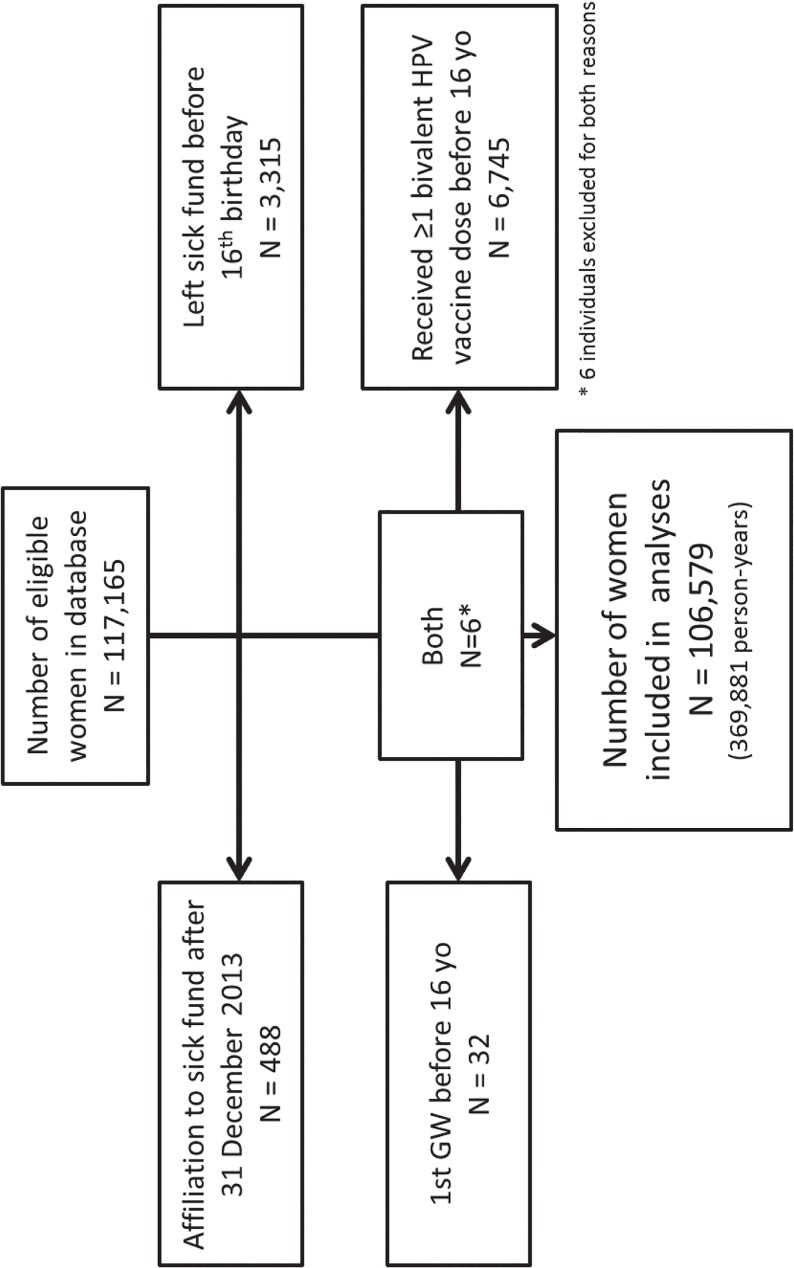
Vaccine effectiveness evaluation. Flow chart summarising the number of women and person-years of follow-up available for the main analysis, with reasons for exclusion.

Overall, 106,579 women were included in the VE analysis, representing 369,881 person-years of follow-up (Fig 2). A total of 35,792 women received three doses of qHPV vaccine; 69.3% (N = 24,791) of them within the recommended schedule (Table 2). The median age at the first qHPV dose was 14.7 years (range: 10.4 to 21.8 years) in women who received three doses. At the end of follow-up, the fully-vaccinated and the unvaccinated women had same median age (20 years).

**Table 2:** Characteristics of the vaccine effectiveness study population as a function of their vaccination status.

Vaccination status	n	Age* at 1st dose (years)	Age* end of study (years)	Number with GWs (%)	Age* 1st GWs episode (years)
Unvaccinated	63 180	-	20.2	244 (0.39)	20.1
1 dose	4 020	15.8	20.1	16 (0.21)	19.1
2 doses	3 587				
3 doses	11 001	14.7	19.7	14 (0.13)	19.1
3 doses (as per schedule)	24 791	14.7	19.9	12 (0.05)	19.3

*Median age

Over the study period, 274 first-time GWs episodes were recorded, 244 among unvaccinated women and 14 among women fully-vaccinated with qHPV vaccine. The median age at first GWs episode was 19.3 years (range: 16.4 to 22.4). The GWs IR by age, parity, HIV status, household income level and region of residence are summarised in Table 3. Overall, the GWs IRs ranged from 21.8 per 100,000 person-years among 16 year-old women to 169.7 per 100,000 person-years among 22 year-old women. GWs IRs seemed higher among women living in Brussels (86.1 per 100,000 person-years), compared with those living in Wallonia or Flanders (71.3 and 66.8 per 100,000 person-years, respectively), but the differences were not statistically significant (Table 3). GWs IR was significantly higher in multiparous compared with nulliparous women. There was no statistically significant difference according to parents’ socioeconomic status (Table 3).

**Table 3:** Estimated genital warts incidence rates per 100 000 person-years, stratified by potential risk factors for the population eligible for vaccine effectiveness assessment.

	Person-years follow-up	No. episodes	Incidence (95% CI) (per 100, 000 person-years)
***Age (years)***			
16	78 072	17	21.8 (13.5; 35.0)
17	73 062	25	34.2 (23.1; 50.6)
18	62 967	49	77.8 (58.8; 103.0)
19	52 710	50	94.9 (71.9; 125.2)
20	42 188	53	125.6 (96.0; 164.4)
21	30 823	37	120.0 (87.0; 165.7)
22	18 855	32	169.7 (120.0; 240.0)
23	6 629	11	165.9 (91.9; 299.6)
Household income*			
High income	321 311	232	72.2 (63.5; 82.1)
Low income	33 325	21	63.0 (41.1; 96.6)
***Region of residence***			
Brussels	65 055	56	86.1 (66.2; 111.9)
Flandre	151 258	101	66.8 (54.9; 81.2)
Wallonie	134 687	96	71.3 (58.4; 87.1)
Outside Belgium	2 768	0	0
***Parity***			
Nulliparous	347 136	234	67.4 (59.3; 76.6)
≥1 birth	18 168	40	220.2 (161.5; 300.1)
***HIV positive***			
No	365 064	274	75.1 (66.7; 84.5)
Yes	241	0	0

* High income: >15,000€ + 3,000€/family member; low income <15,000€ + 3,000€/family member

The GWs IR was 111.7 (95% CI: 98.5; 126.6) per 100 000 person-years in unvaccinated women compared with 12.0 (95% CI: 7.1; 20.3) per 100 000 person-years in fully-vaccinated women (Table 4). The crude qHPV VE against GWs was 89.2% (95% CI: 81.5; 93.7) for a completed vaccination schedule and 87.2% (95% CI: 77.2–92.8), when the three doses were given according to schedule (Table 4). The crude VE remained higher than 80%, for over four years post-vaccination (Table 4). Crude qHPV VE was 36.9% (95% CI: -15.5; 65.5) after one dose and 69.7% (95% CI: -26.6; 87.5) after two doses.

**Table 4:** Crude and age-adjusted vaccine effectiveness (VE) estimates for genital wart by number of qHPV vaccine doses, time since vaccination and age at first vaccine dose. Women eligible for HPV vaccination in Belgium and affiliated to the MLOZ sick fund.

Vaccination status	Person-years of follow-up	Number of episodes	Incidence per 100000 (95% CI)	Crude VE (95% CI)	Age-adjusted VE (95% CI)
Unvaccinated	218 524	244	111.7 (98.5; 126.6)		
1 dose	15 608	11	70.5 (39.0; 127.3)	36.9 (-15.5; 65.5)	36.6 (-16.1; 65.4)
2 doses	14 794	5	33.8 (14.1; 81.2)	69.7 (26.6; 87.5)	65.7 (16.9; 85.9)
1 or 2 doses	30 402	16	52.6 (32.2; 85.9)	52.9 (21.8; 71.6)	49.8 (16.7; 69.8)
Fully vaccinated	116 379	14	12.0 (7.1; 20.3)	89.2 (81.5; 93.7)	88.0 (79.4; 93.0)
Fully vaccinated*	84 074	12	14.3 (8.1; 25.1)	87.2 (77.2; 92.8)	85.9 (74.8; 92.1)
Fully vaccinated since:					
30 days- ≤1 year	14 301	3	21.0 (6.8; 65.0)	81.2 (41.3; 94.0)	66.4 (-6.0; 89.3)
>1 year- ≤2 years	22 668	1	4.4 (0.6; 31.3)	96.0 (71.8; 99.4)	99.7 (-54.8; 99.1)
>2 years-≤4 years	52 876	7	13.0 (6.3; 27.8)	88.1 (74.9; 94.4)	86.9 (72.2; 93.9)
>4 years	26 534	3	11.2 (3.6; 35.1)	89.9 (68.4; 96.8)	92.2 (75.5; 97.5)
Fully vaccinated with first dose:					
at <15 years	57 595	5	8.7 (3.6; 20.9)	0.1 (0.0; 0.3)	89.0 (73.2; 95.5)
between 15 and 17 years	53 149	6	11.3 (5.1; 25.1)	0.1 (0.0; 0.2)	90.4 (78.3; 95.7)
at ≥18 years	5 636	3	53.2 (17.2; 165.0)	0.3 (0.1; 1.0)	68.5 (1.2; 89.9)

* fully vaccinated according to schedule

In the multivariate analyses, after excluding variables with p≥0.10, only age remained independently associated with the outcome and was included as an adjustment factor in the final Poisson regression model. Age-adjusted qHPV VE against GWs was 88.0% (95% CI: 79.4; 93.0) in fully vaccinated women (Table 4). The age-adjusted qHPV VE was similar to the crude VE for the various schedules of vaccination, ranging from 36.6% (95% CI: -16.1; 65.4) for a single dose to 88.0% (95% CI: 79.4–93.0) for a full schedule (table 4). The age-adjusted qHPV VE was higher when the first dose was given to women <18 years (Table 4).

The results obtained in the various sensitivity analyses were similar to those in the main analyses. When including all women aged 16 to 23 years, regardless of their eligibility for qHPV vaccination, the age-adjusted qHPV VE was 50.8% (95% CI: 22.2; 68.9) for partially vaccinated women and 89.0% (95% CI: 81.3; 93.6) for fully-vaccinated women.

Before performing the second sensitivity analysis, we verified that women vaccinated with bHPV vaccine had a similar GWs IR as women who had not received any HPV vaccine. We found GWs IRs of 111.7 (95% CI: 98.5; 126.6) and 128.6 (95% CI: 86.2; 191.9) per 100,000 person-years, respectively, in unvaccinated women and in women who had received three doses of bHPV vaccine (data not shown), enabling these two groups to be merged. In this second sensitivity analysis, the GWs IR was 103.5 (95% CI: 92.1; 116.3) per 100,000 person-years in the non-qHPV vaccine group vs 14.2 (95% CI: 8.8; 22.8) per 100,000 person-years among women fully vaccinated with qHPV vaccine. The age-adjusted qHPV VE was 88.4% (95% CI: 80.1; 93.2).

## Discussion

We observed an important qHPV VI in the population targeted by the vaccination programme (i.e. women aged 16 to 22 years) with an observed decrease in GWs IR of 72.1% (95% CI: -77.9; -64.7) between pre and post-vaccination periods; GWs IR decreased from 257.3 per 100 000 (pre-vaccination; 2006) to 71.5 per 100 000 (post-vaccination; 2009–2013) for these women. The significant decreases in GWs IRs observed in the youngest men, aged 16 to 22 years (-51.1%) and in women aged 23 to 30 years (-18.8%) suggest the presence of herd-protection. However, we cannot exclude that some males and older women may have been vaccinated with the qHPV vaccine without reimbursement. Although, a statistically significant decline of 8.1% in GWs IR was observed in the overall population, we did not find any evidence of herd-protection in other age groups.

These results are consistent with those observed in other observational studies evaluating the VI of qHPV vaccination programmes on GWs. Since the first study from Australia that reported a reduction in GWs incidence after the introduction of qHPV vaccine, many studies have also reported early VI, with a similar herd effect in young males in most of them [22–24, 26–30]. Despite the fact that these studies were performed using different data sources and in settings with different levels of qHPV vaccine uptake, the results consistently demonstrate an early impact on GWs incidence in the population targeted by the qHPV vaccination programmes.

The observed increased GWs IRs in older age groups is consistent with data from other countries, for example Sweden and Germany [22, 26]. This increase could reflect changes in sexual behaviour, a phenomenon that is supported by the reported increased incidence of other sexually-transmitted infections (STIs), such as chlamydia, in Belgium and Australia [31, 32].

The high qHPV VE we observed in fully-vaccinated women is consistent in all sensitivity analyses. It is also consistent with the high vaccine efficacy estimates observed in clinical trials and VE estimates observed in other observational studies [18, 25]. The observed qHPV VE was lower for those who did not received all three doses of qHPV vaccine. The observed median interval between doses was 63 days (about 2 months) among those who received two doses which is more likely to be representative of an ‘incomplete’ three-dose schedule (i.e. only the first two doses of the three-dose schedule), than of the recently recommended 2-dose schedule, where an interval of at least 6 months is recommended between the two doses in individuals aged 9 to 13, to provide full protection [33]. Hence the lower VE reported with two doses compared with three doses, in this context, is not unexpected.

The qHPV VE was higher in women who were vaccinated at a younger age (before 18 years-old), possibly because these women are less likely to have been exposed to HPV before vaccination. Recently, the median age at first sexual intercourse in Belgium has been reported to be 17.2 years [32]. Since qHPV vaccine is a prophylactic vaccine, it can prevent the acquisition of new HPV infections but it does not treat existing infections. The vaccine is thus more effective when given before sexual debut or in women who are HPV naïve at vaccination. These results were expected and are consistent with those observed in other studies and with the results from clinical trials where it has also been reported that the younger the age of vaccination, the higher the vaccine efficacy [34, 35]. Finally, our data suggest a sustained qHPV VE over time, with an observed VE >80% for more than four years (Table 4).

Our study was performed in a large database, which allowed us to link individual vaccination reimbursement data with a specific surrogate marker for GWs [17]. This enabled us to evaluate both the qHPV VE at the individual level and the VI of the HPV vaccination programme at a population level. Also, we were able to assess the qHPV VE as a function of the number of doses, adherence to the recommended schedule, age at first dose and time since vaccination.

Our study presents some limitations, the main being that a surrogate marker was used to evaluate the incidence of GWs. However, this surrogate marker is highly likely to be specific for GWs since an agreement has to be obtained from the sickfund physician prior to reimbursement, and the reimbursement level for imiquimod for treating GWs is different than that for its other indications. This approach may have resulted in an underestimation of the true GWs incidence and, as a consequence, in an underestimation of the VI and VE for a first GWs episode. It is possible that individuals with GWs may not have consulted their physician for treatment during the study period or that other treatments were used, although it has been estimated that imiquimod is used to treat about 70% of GWs episodes in Belgium [17].

Changes in GWs treatment approaches over the study period could also have affected the VI estimate. In Belgium, imiquimod has been reimbursed for GWs treatment since 2002 and the number of prescriptions increased afterwards. To minimise the influence of changes in imiquimod use during the study period, we used 2006 as the reference year, assuming that prescription had stabilised at that time, even if it would have been better to have trends for imiquimod prescription before vaccine introduction for a longer period. However, as recommendations for GWs treatment in Belgium did not change over study period, we assumed that imiquimod prescriptions in 2006 would be an acceptable baseline for VI evaluation.

HPV vaccine introduction may have been accompanied by health promotion messages on safer sexual practices, which could have increased the measured VI by decreasing risk behaviour in the study population. If this were the case, the incidence of other STIs would have also declined. However, this was not the case, since recent data from the Belgian Institute of Public Health, show that the incidence of several STIs increased during the study period [36]. For example, chlamydia increased in all age-groups, in particular, in men and women aged 20–24 years. We cannot exclude the fact that the introduction of qHPV vaccine may have influenced sexual behaviour and may have resulted in different infection risks between vaccinated and unvaccinated women and thus affect the estimated VE. Either vaccinated women could have felt more protected, resulting in increased risk-taking behaviour, or they could have felt more concerned about their sexual health and used condoms to prevent other STIs more regularly than unvaccinated women. These different behaviours would lead to an overestimation and underestimation, respectively, of the GWs IR in vaccinated women with a consequent underestimation and overestimation, respectively, of the VE.

Over the study period, it is possible that the age distribution of the affiliates varied. This could have changed the risk of GWs, if more individuals were younger. To minimize any potential bias from variation in the age distribution among the affiliates over the years, age-standardized calculations were used.

Other factors likely to have affected VE evaluation are related to usual limitations of database analyses. Firstly, the reimbursement for qHPV vaccine was used as a surrogate for vaccine exposure. About 1,000 of the 43,399 women who had received ≥1 qHPV vaccine dose had more than three reimbursements during the study period. This could be because one or more doses were lost, some women may have received more than three doses, or the doses could have been administered to another person, who may have, therefore, been erroneously considered to be unvaccinated in our study. In addition, some women, and possibly some men may have been vaccinated without reimbursement. These misclassifications in exposure would have resulted in an underestimation of VE. Secondly, the date of vaccine reimbursements and imiquimod agreements were used as the date of vaccination and GWs onset, respectively, resulting in inaccurate dates for vaccine exposure and particularly for onset of GWs, as the real delay between GWs diagnosis and agreement for treatment may be as long as three to six months. This could have resulted in an overestimation of the GWs IR in vaccinated women and thus an underestimation of the VE.

Although we included a large number of women in the VE analysis, there were relatively few new GWs episodes, so that the results from the stratified analyses should be interpreted with caution. They should be taken as being suggestive of trends that should be confirmed, rather than as conclusive results.

High HPV vaccine coverage rate is a key factor for the success of the qHPV vaccination programme in the prevention and control of GWs, CIN and cervical cancer. Since we performed this study, Belgium has changed to a school-based vaccination programme which is expected to increase vaccine coverage. In addition to monitoring safety, monitoring VI and VE in a real-life setting provides important data for the benefit/risk ratio evaluation, which can help to inform vaccine policies and contribute to the improvement of vaccination programmes.

In conclusion, we observed early, substantial and sustained real-life benefits on the incidence of GWs following qHPV vaccine introduction in Belgium, both on population and individual levels. The potential biases associated with the study design, are likely to have mainly resulted in an underestimation of the benefit. Highest VI estimates were observed in the population targeted by the HPV vaccination programme. The high VE estimates are consistent with the efficacy results from clinical trials and VE estimates from other observational studies conducted in different settings. Our results also suggest a herd protection for younger men and that vaccination at a younger age offers better protection against genital warts. Continual evaluation of the benefits from the qHPV vaccination programme is essential to verify that these promising early benefits are sustained.

## Data Sharing Statement

Sanofi-Pasteur MSD (SPMSD) is not the owner of the database extracted for these reported analyses. These data were provided to SPMSD by MLOZ for the purpose of the study analyses only. Therefore, SPMSD cannot provide access to the data directly. Requests for raw data extractions can be addressed to Rudy Van Tielen, MLOZ (Rudy.VanTielen@mloz.be). This does not alter our adherence to PLOS ONE policies on sharing data and materials.
