# Correction: Evaluating the Early Benefit of Quadrivalent HPV Vaccine on Genital Warts in Belgium: A Cohort Study

**DOI:** 10.1371/journal.pone.0148665

**Published:** 2016-01-29

**Authors:** Geraldine Dominiak-Felden, Corrado Gobbo, François Simondon

The image for Fig 1 is incorrect. Please see the correct Fig 1 here.

**Fig 1:**
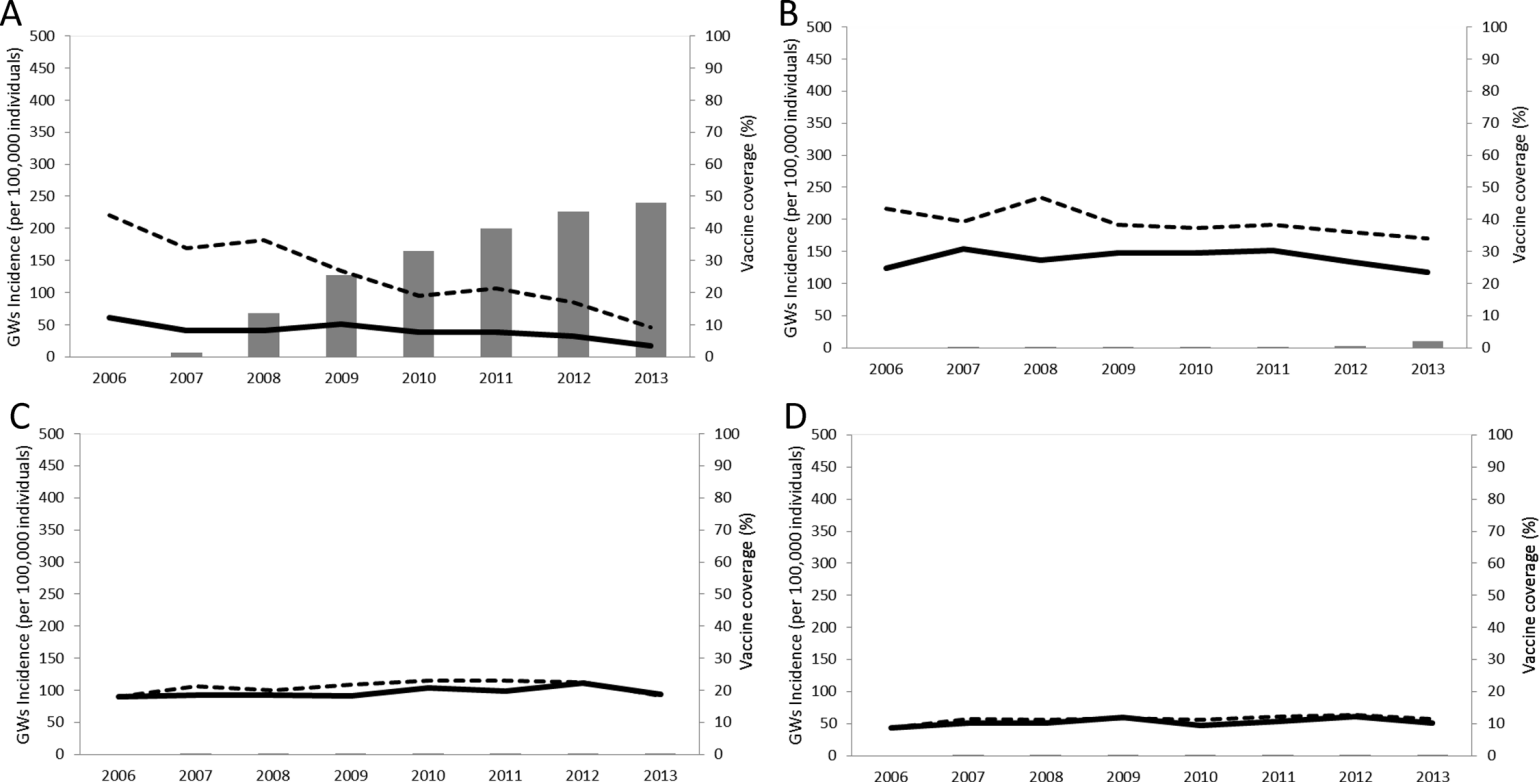
Incidence of genital warts per 100 000 individuals (dashed line: females; solid line: males) and qHPV vaccine uptake in individuals aged 16–22 years (A), 23–30 years (B), 31–40 years (C) and 41–59 years (D) affiliated to the MLOZ sick fund (Belgium) between 2006 and 2013 (standardised estimates).

There are errors in the fourth row of Table 2. Please see the corrected Table 2 here.

**Table 2:** Characteristics of the vaccine effectiveness study population as a function of their vaccination status.

Vaccination status	n	Age* at 1^st^ dose (years)	Age* end of study (years)	Number with GWs (%)	Age* 1^st^ GWs episode (years)
Unvaccinated	63 180	-	20.2	244 (0.39)	20.1
1 dose	4 020	15.8	20.1	16 (0.21)	19.1
2 doses	3 587				
3 doses	35 792	14.7	19.7	14 (0.04)	19.1
3 doses (as per schedule)	24 791	14.7	19.9	12 (0.05)	19.3

*Median age

There is an error in the third sentence of the last paragraph of the Results section. The correct sentence is: In this second sensitivity analysis, the GWs IR was 113.9 (95%CI: 101.3–128.1) per 100,000 person-years in the non-qHPV vaccine group vs 14.2 (95% CI: 8.8; 22.8) per 100,000 person-years among women fully vaccinated with qHPV vaccine.
